# Antimicrobial Films Based on Nanocomposites of Chitosan/Poly(vinyl alcohol)/Graphene Oxide for Biomedical Applications

**DOI:** 10.3390/biom9030109

**Published:** 2019-03-18

**Authors:** Sebastián Ruiz, Julián Andrés Tamayo, Johannes Delgado Ospina, Diana Paola Navia Porras, Mayra Eliana Valencia Zapata, José Herminsul Mina Hernandez, Carlos Humberto Valencia, Fabio Zuluaga, Carlos David Grande Tovar

**Affiliations:** 1 Escuela de Ingeniería de Materiales, Facultad de Ingeniería, Universidad del Valle, Calle 13 No. 100-00, Santiago de Cali 760032, Colombia; sebastian.ruiz.londono@correounivalle.edu.co (S.R.); julian.tamayo@correounivalle.edu.co (J.A.T.); valencia.mayra@correounivalle.edu.co (M.E.V.Z.); jose.mina@correounivalle.edu.co (J.H.M.H.); 2 Grupo de Investigación Biotecnología, Facultad de Ingeniería, Universidad de San Buenaventura Cali, Carrera 122 # 6-65, Cali 76001, Colombia; jdelgado1@usbcali.edu.co (J.D.O.); dpnavia@usbcali.edu.co (D.P.N.P.); 3 Escuela de Odontología, Grupo Biomateriales Dentales, Universidad del Valle, Calle 13 No. 100-00, Cali 76001, Colombia; carlos.humberto.valencia@correounivalle.edu.co; 4 Laboratorio SIMERQO, Departamento de Química, Universidad del Valle, Calle 13 No. 100-00, Cali 76001, Colombia; hector.zuluaga@correounivalle.edu.co; 5 Programa de Química, Facultad de Ciencias, Universidad del Atlántico, Carrera 30 No. 8-49, Puerto Colombia 081008, Colombia

**Keywords:** biodegradable films, chitosan, graphene oxide, poly(vinyl alcohol), tissue engineering

## Abstract

Today, tissue regeneration is one of the greatest challenges in the field of medicine, since it represents hope after accidents or illnesses. Tissue engineering is the science based on improving or restoring tissues and organs. In this work, five formulations of chitosan/poly(vinyl alcohol)/graphene oxide (CS/PVA/GO) nanocomposites were studied for the development of biodegradable films with potential biomedical applications. The characterization of the films consisted of Fourier-transform infrared (FTIR) spectroscopy, scanning electron microscopy (SEM) and energy dispersive spectroscopy (EDS). The antibacterial activity was evaluated in vitro against Gram-positive bacteria *Bacillus cereus* and *Staphylococcus aureus* and Gram-negative *Salmonella* spp. and *Escherichia coli*, by contact of the film above inoculum bacterial in Müeller–Hinton agar. On the other hand, in vivo tests in which the material implanted in the subcutaneous tissue of Wistar rats demonstrated that the formulation CS/PVA/GO (14.25:85:0.75) was the best antibacterial film with adequate degradation in vivo. All together, these results indicate the potential of the films using nanocomposites of CS/PVA/GO in tissue engineering and cell regeneration.

## 1. Introduction

Chitosan (CS) is a natural biopolymer derived from the deacetylation of chitin obtained from crustacean shells which can be processed to form films, fibers, beads, and dust [1,2]. Chitosan has become an attractive polymer for biomedical applications due to its antimicrobial properties and biocompatibility [3], with applications in tissue engineering of bone, cartilage, liver, tendons, ligaments and nerves, in wound dressings, separation membranes, blood anticoagulants, contact lenses, controlled release of drugs, as a fat-sequestering agent, in hydrogels and as food packaging material [4,5,6,7]. However, the high amount of hydrogen bonds of the amino and hydroxyl groups in the CS has created some inherent disadvantages, such as low mechanical properties, poor solubility in conventional solvents as well as poor stability in physiological media [8,9,10,11].

Many studies are addressing the physicochemical attributes of hybrid polymers (natural and synthetic origin), thanks to their effectiveness in overcoming the disadvantages of the naturally occurring polymer [12]. Among the fillers used are ceramic nanoparticles [13], oxides [14], and cross-linking agents [15], among other materials. However, polyvinyl alcohol/chitosan (PVA/CS) compounds are traditionally used to prepare films with improved properties, because PVA is biocompatible, which allows its use in clinical applications, membrane fabrication and tissue repair [16].

Thanks to its high mechanical resistance, chemical stability, large surface area, and low toxicity [17,18], in recent years graphene oxide (GO) has had rapid growth in biomedical applications [19]. The presence of polar groups on the surface of this material improves compatibility with polymer matrices, such as CS [20]. Also, due to its antibacterial properties and its high surface/volume ratio, the GO is a promising material for the development of antimicrobial and antiviral surfaces [21].

Although several studies on CS/PVA/GO films are in the literature, there is a lack of works where the characterization includes the physical, chemical, and mechanical evaluation of the films, together with biological tests in vitro and in vivo conditions. Therefore, this research aimed to study biodegradable films from PVA/CS formulations reinforced with GO nanofilms grouping all this characterization.

## 2. Materials and Methods

### 2.1. Materials

For GO synthesis, graphite flakes (99.8%) were used (Alfa Aesar, Tewksbury, MA, USA). Concentrated sulfuric acid (H_2_SO_4_), potassium permanganate (KMnO_4_), hydrogen peroxide (H_2_O_2_) and isopropanol were supplied from Merck (Burlington, MA, USA). For the production of the films, CS of low molecular weight (144,000 g/mol) and a deacetylation degree between 89% and 90%, PVA with hydrolysis between 87% and 89% and viscous molecular weight of 93,000 g/mol (Sigma-Aldrich, Palo Alto, CA, USA). Glacial acetic acid was purchased from Merck. For the elaboration of the simulated biological fluid, NaCl, K_2_HPO_4_ ·3H_2_O, CaCl_2_, Na_2_SO_4_, tris-(hydroxymethyl aminomethane) [(CH_2_OH)_3_CNH_2_] were acquired from Sigma-Aldrich; NaHCO_3_, KCl, MgCl_2_ ·6H_2_O from Fisher Chemical (Pittsburgh, PA, USA) and hydrochloric acid (HCl) from Merck. All reagents used were analytical grade.

### 2.2. Methods

#### 2.2.1. Graphene Oxide Synthesis

Graphene oxide synthesis followed the methodology used by Mangadlao et al. [22]. In a round flask, 3 g of graphite were mixed with 400 mL of concentrated H_2_SO_4_, after 10 min of stirring, 3 g of KMnO_4_ were added. Subsequently, 3 g of KMnO_4_ were added every 24 h for three days. The reaction was stopped at four days, for which a third of the contents of the balloon was mixed in 300 mL of a water/ice mixture and 2 mL of H_2_O_2_ were added. The solution was distributed in 50 mL flasks and washed in a UNIVERSAL 320R centrifuge (Hettich, Tuttlingen, Germany) for 10 min at 5000 rpm with Milli-Q water and isopropanol until reaching a neutral pH. Subsequently, it was purified by dialysis in 3.5K MWCO SnakeSkin tubes (Thermo Scientific, Waltham, MA, USA), followed by Milli-Q water washing and drying at −51 °C and 0.12 mBar pressure in a Freezone 4.5 freeze dryer (LABCONCO, Kansas City, MO, USA) for 48 h. The graphite oxide was dispersed in water for 24 h in a Branson 5800 ultrasound equipment (Branson, Madrid, Spain) to finally obtain the GO.

#### 2.2.2. Graphene Oxide Characterization

The GO and the starting graphite were chemically characterized by Fourier-transform infrared (FTIR) spectroscopy on an IR Affinity-1 infrared spectrophotometer (Shimadzu, Kyoto, Japan) in a range of 500–4000 cm^−1^ in transmittance mode using the KBr pellet method. The X-ray spectra were taken on a Panalytical X’PERT PRO diffractometer (Malvern, Royston, UK) using Cu Kα1 radiation (1.540598 Å) and Kα2 (1.544426 Å), with an electron accelerator voltage of 45 kV, an electron-generating current of 40 mA, an optical grid of incident beam 1° and 1/2° and a diffracted beam grid of 9.1 mm, in a range 2θ between 5° and 40°. We used the Bragg law (Equation (1)) to determine the distance between two planes of the GO network:

(1) $$d=\frac{\lambda }{2sen\theta }$$

where *d* is the lattice spacing, *λ* is the X-ray wavelength and *θ* is the angle of incidence.

The characteristic bands of GO and graphite were acquired by Raman spectroscopy in a Thermo Scientific X-ray diffraction (XRD) equipment (Thermo Scientific), with an excitation source of 512 nm at room temperature.

#### 2.2.3. Film Preparation

The films were prepared according to the film casting method described by Liu et al. [23], which consisted of clearing out the solutions described in Table 1, in acetate molds and subsequently, letting cure for 24 h in the environment, and then 24 h in the oven to a temperature between 38 and 40 °C, thus obtaining the solid film of CS/PVA/GO. Once collected, the test specimens were cut and conditioned for the tensile test according to ASTM D6287 and ASTM D618 standards with the dimensions specified in ASTM D882. The thickness of each specimen was determined using a Mitutoyo digital micrometer No. 293-330 (Kawasaki, Japan), from three average values along each sample. The samples were placed in a desiccator at 10% relative humidity (RH) until the time of the test.

For the preparation of the solutions, CS was dissolved in a 2% (w/v) acetic acid solution for 12 h at 80 rpm. The PVA was dissolved in distilled water for 3.5 h at 80 °C and 300 rpm, and finally, the GO was dispersed in distilled water using an ultrasonic bath (Branson) for 24 h. The formulations used in this research are in Table 1.

After the solutions were prepared separately, CS and PVA were mixed by stirring at 90 rpm for 2 h until a homogeneous solution was obtained which was filtered and finally, the GO dispersion was added with stirring until a homogeneous solution was obtained.

#### 2.2.4. Film Characterization

##### Fourier-Transform Infrared Spectroscopy

The chemical identification of the films was carried out using FTIR in the attenuated total reflectance (ATR) mode (Shimadzu).

##### Scanning Electron Microscopy

The morphological inspection of the surfaces of the film was carried out by scanning electron microscopy (SEM) (JEOL JSM-6490LA, Musashino, Tokyo, Japan). The working conditions were 20 kV and mode of secondary backscattered electrons. All the samples were coated with gold, to create an electronic density in the material, since the polymers lack it.

##### The Tensile Strength of Films

For the tensile test, a universal SHIMADZU EZ-LZ test machine (Shimadzu) was used, following the ASTM D882 standard. Where at least five samples per formulation are required, the gap between jaws was 100 mm, the width of the film was 20 mm, and the test speed was 50 mm/min.

##### Degradation in Simulated Biological Fluid

The hydrolytic degradation was carried out following the procedure outlined in the ASTM F1635-16 standard. The films were immersed in a simulated biological fluid (SBF) at 37 °C for seven days in a Memmert IN 110 incubator (Memmert GmbH & Co. KG, Schwabach, Germany). The SBF was prepared according to the method proposed by Kokubo and Takadama [24] and the degradation was evaluated by examining the weight of the films before and after immersion for different periods (one, three, five, and seven days).

The initial weight of the samples before immersion was recorded as $W_{0}$ and the weight after drying for 48 h in the incubator at 37 °C was recorded as $W_{d}$. The weight loss (% $W_{l}$) was calculated according to Equation (2):

(2) $$W_{l}\;\left(\%\right)=\frac{W_{0}-W_{d}}{W_{0}}\times 100$$

Each sample was immersed in 15 mL of SBF, and in each period three samples were evaluated per formulation. The pH of the SBF was measured every day until the total test time was completed using an Fisherbrand™ accumet™ AB150 pHmeter (Fischer Scientific, Inc., Ottawa, Canada). The morphology of the films after drying was studied using SEM.

##### Antimicrobial Film Assay

The antimicrobial activity of the films was evaluated against bacteria *Bacillus cereus*, *Staphylococcus aureus*, *Salmonella* spp., and *Escherichia coli*, by contact of the film above inoculum bacterial in agar. In short, the inoculum of each of the bacteria was carried out by adding 40 μL of dilution to 10^−6^ colony forming units (CFU) of each of the 24 h growth strains in 90 mm diameter Petri dishes containing 20 mL of Müeller–Hinton agar. Subsequently, a disc with 10 mm of diameter of each of the prepared films was placed in the center of the box on the inoculated agar. The plates with the strains were incubated at 37 °C, and the reading of the results was carried out 24 h after the inoculation. The inhibition was determined as the absence of growth observed between the transparent film and the previously inoculated agar. The test was repeated three times for each of the treatments.

##### Biomodel Tests In Vivo

The inflammatory response to the implantation of films in the subcutaneous tissue. Samples of CS/PVA films with different percentages of GO (three replicates per formulation) with 10 mm in diameter and 2 mm in thickness were implanted in subdermal tissue of three adult Wistar rats, in preparations made on the dorsal surface, according to the recommendation of ISO 10993-6 and as reported previously [25]. As a control sample, commercial porcine collagen BioMend® (ZIMMER BIOMET, Miami, FL, USA) with the same dimensions was used. All the biomodels were supplied by the Bioterio of the Faculty of Medical Sciences of the Universidad del Valle. The procedures carried out were approved by the Animal Ethics Committee of Universidad del Valle by the CEAS 001-016 certificate.

After 30 days of implantation, the samples were recovered, fixed in buffered formalin, dehydrated in alcohol solutions of ascending concentration (70%, 80%, 95%, and 100%), diaffinized with xylol and infiltrated with paraffin for later cutting at 4 µm using a Thermo ScientificTM Histoplast Paraffin ™ and an Autotechnicon Tissue Processor ™ (Leica Microsystems, Mannheim, Germany). The samples were processed for histological analysis by hematoxylin and eosin and Masson trichromacy techniques. For the analysis of the images, a Leica DM 750 microscope with a Leica DFC 295 camera and Leica Application Suite version 4.12.0 (Leica Microsystems, Mannheim, Germany) imaging software was used. The macroscopic images were taken with a Samsung DualView DV150F 16 MP digital camera.

## 3. Results and Discussion

### 3.1. Graphene Oxide Characterization

#### 3.1.1. Fourier-Transform Infrared Spectroscopy

**Figure 1:**
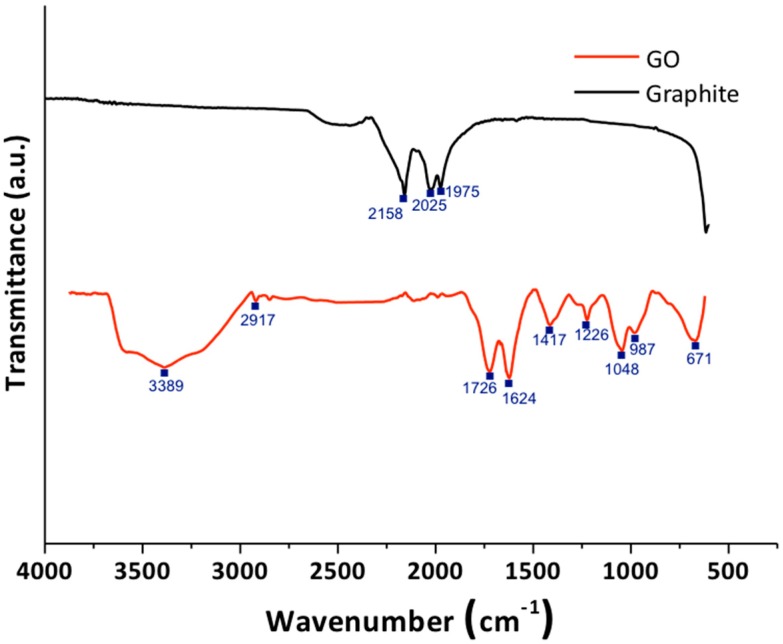
Fourier-transform infrared (FTIR) spectroscopy of graphite (black line) and graphene oxide (GO; red line). a.u.: Arbitrary units.

Figure 1 shows the FTIR spectrum of graphite and graphene oxide. For the graphite, it exhibits few absorption signals, due to the difference in the state of charges between carbon atoms. This weak difference leads to a very small induced electric dipole, which provides a very clean spectrum [26]. It is clear that when an oxidation treatment is used on the material, bands corresponding to oxygenated functional groups appear, which indicates that the oxidative process was successful. The graphene oxide spectrum showed the band at 3389 cm^−1^, which corresponds to the stretching vibration of the hydroxyl groups (O–H). The band at 2917 cm^−1^ corresponds to the vibration of the C–H bond that is in sp^3^ hybridization. The band at 1726 cm^−1^ corresponds to the stretching vibration of the carbonyl group (C=O). The band at 1624 cm^−1^ corresponds to the stretch mode of the sp^2^ carbon skeletal network (C=C). It is also evident, according to some results reported in the literature, that the GO obtained contains several oxygen functional groups, such as hydroxyl, carboxyl, and epoxy groups, which correspond to the bands presented at 1417, 1226, 1048, 987, and 671 cm^−1^ [25,27,28].

#### 3.1.2. X-Ray Diffraction

**Figure 2:**
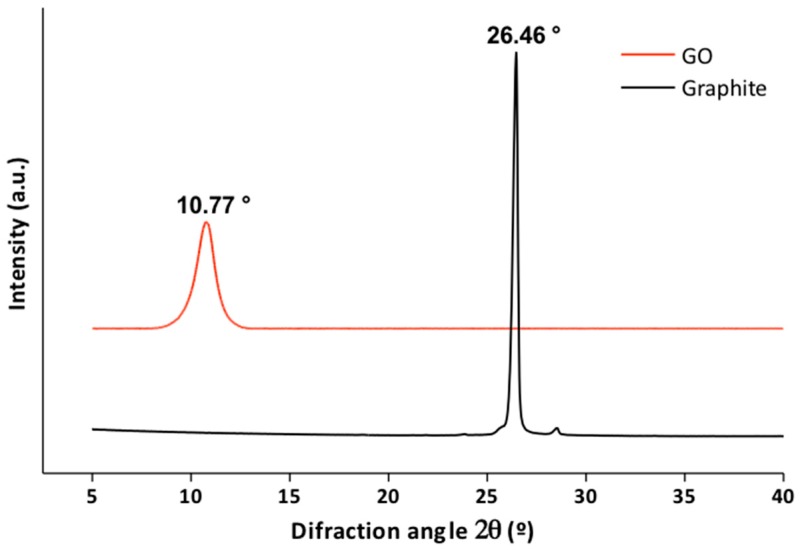
X-ray diffraction (XRD) of graphite (black line) and graphene oxide (GO; red line).

Figure 2 shows the X-ray diffraction (XRD) pattern of graphite and GO. The graphite exhibits a peak at 2θ = 26.46° which corresponds to the reflection of the plane (002), while for the GO the peak appears at 2θ = 10.77° and corresponds to the reflection of the plane (001) [29,30,31,32,33]. The increase in the interplanar distance of the GO in comparison with the graphite is shown in Table 2 and due to the presence of functional oxygen groups introduced by the oxidation of the graphite, which facilitate the exfoliation and hydration of the nanofilm of GO in an aqueous solution [30]. This increase in interplanar distance has been widely reported. The characteristic spacing of the GO generally ranges between 0.7 and 0.8 nm but may vary slightly at higher or lower values, depending on the degree of functionalization [30,34,35].

#### 3.1.3. Raman Spectroscopy

**Figure 3:**
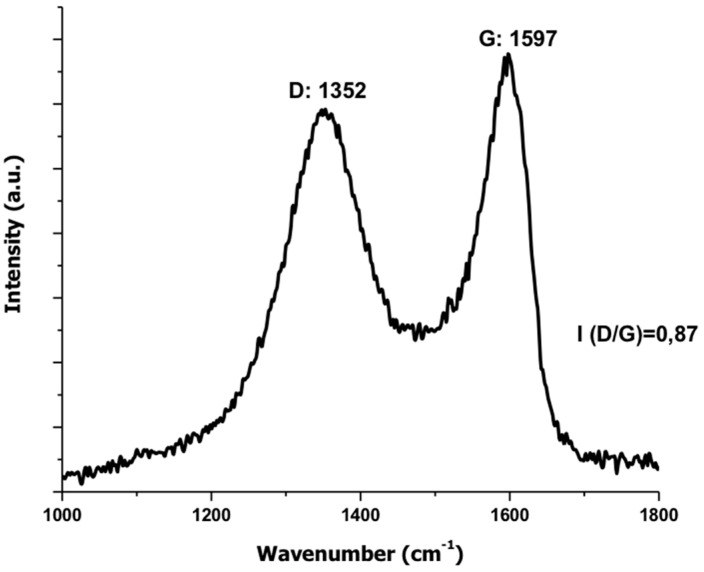
Raman spectrum of graphene oxide.

According to the Raman spectrum for the GO shown in Figure 3, the G peak attributed to the stretching of the bond of the carbon pairs in sp^2^ hybridization, and the peak D associated with the breathing mode of the sp^2^ carbon rings and activated by defects caused in the graphite network due to the presence of oxygen-rich functional groups [36]. The relationship between the intensities of bands D and G is directly related to the degree of oxidation of the GO sheets [37].

### 3.2. Film Characterization

#### 3.2.1. Fourier-Transform Infrared Spectroscopy 

**Figure 4:**
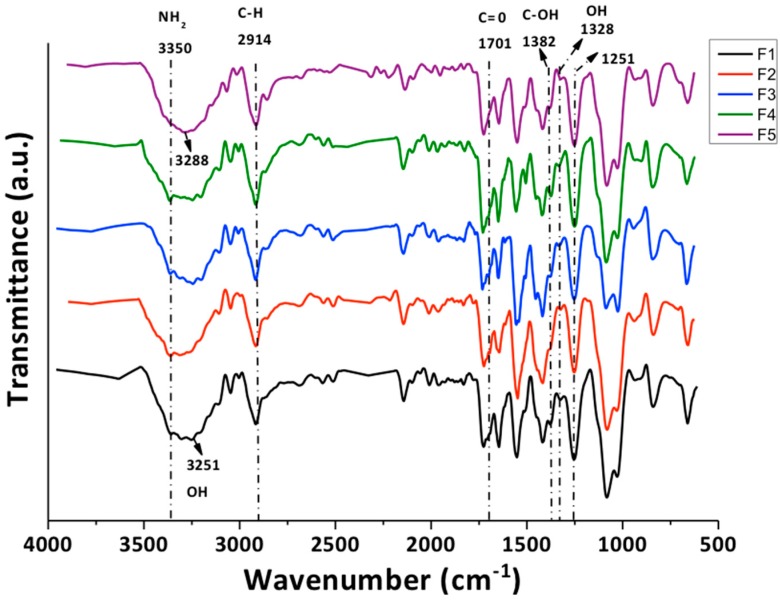
Attenuated total reflectance Fourier-transform infrared (ATR-FTIR) spectroscopy of chitosan/poly(vinyl alcohol)/graphene oxide (CS/PVA/GO) films for the five formulations. F1: CS/PVA/GO 20:80:0; F2: CS/PVA/GO 14.75:85:0.25; F3: CS/PVA/GO 19.5:80:0.5; F4: CS/PVA/GO 14.25:85:0.75 and F5: CS/PVA/GO 29:70:1. All the ratios are wt %.

In Figure 4 it is observed that an absorption band appears at 1701 cm^−1^ related to the stretching vibration of C=O carboxyl groups at the GO edges. The band of vibration associated with the –OH group (1328 cm^−1^) becomes more pronounced with the increase of the GO content due to the destruction of the original hydrogen bonds of CS/PVA compounds and the formation of a strong interaction between CS, PVA, and GO. The C–OH bond, observed at 1382 cm^−1^, is weakened due to the increase in GO due to the strong hydrogen bond. Besides, the band at 3251 cm^−1^ (stretching vibration of the –OH group) was displaced to higher values (3288 cm^−1^) and was further extended, due to the interaction of the GO with the CS/PVA mixture by hydrogen bonds [35].

#### 3.2.2. Scanning Electron Microscopy

**Figure 5:**
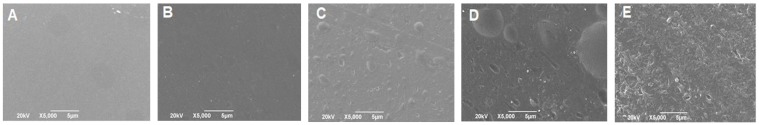
Morphology of CS/PVA/GO films. (**A**) F1: CS/PVA/GO 20:80:0; (**B**) F2: CS/PVA/GO 14.75:85:0.25 ); (**C**) F3: CS/PVA/GO 19.5:80:0.5; (**D**) F4: CS/PVA/GO 14.25:85:0.75; (**E**) F5: CS/PVA/GO 29:70:1. All the ratios are wt %.

As shown in the SEM micrographs (Figure 5), an increase in the roughness of the film surface is evidenced in the different formulations, the morphology of the films changes from a smooth structure for formulation F1 (F1; Figure 5A), to an utterly rough morphology for formulation F5 (F5; Figure 5E). Previous studies have shown that the surface of pure CS and PVA films separately is smooth, continuous and compact [16,38], so it is expected that the mixture between them will also present a surface with the same conditions, which is consistent with the results found, because the PVA offers good compatibility when incorporated into polysaccharides such as CS. By adding GO to the CS/PVA mixture, the GO nanosheets generate an increase in the roughness of the films due to their texture [35]. There is also a specific porosity which is possibly associated with the rapid evaporation of the solvent during the curing of the films, air bubbles or separation of polysaccharide molecules during the curing process of the films [16].

In general, CS/PVA films were transparent, which can be explained by the inherent property of PVA and the amorphous nature of chitosan [35]. With GO content increasing, there was an increase in the intensity of the black tone and consequently an increase in the opacity of the film [39].

#### 3.2.3. The Tensile Strength of the Films

**Figure 6:**
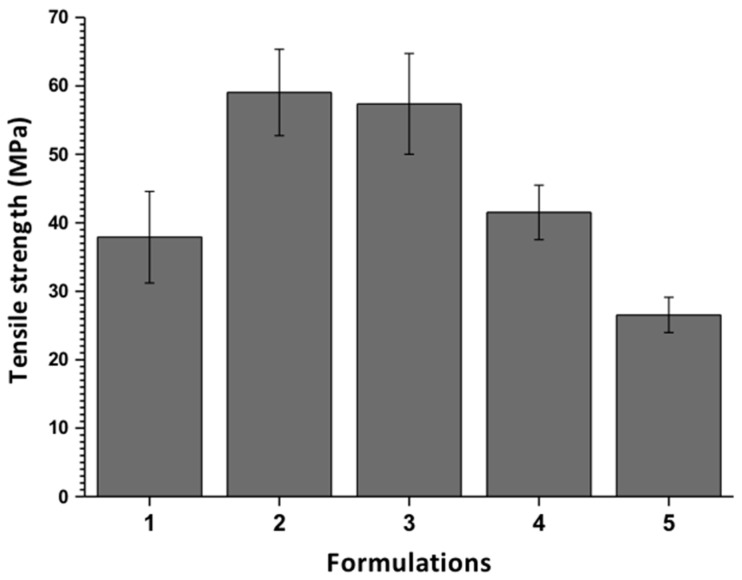
The tensile strength of CS/PVA/GO films. Data are shown as mean ± standard deviation of the mean (*n* = 5).

As shown in Figure 6, the tensile strength is affected by the fraction of each component. It is evident that the presence of GO in percentages lower than 1 wt % improves the traction properties of the films. It is evident that the incorporation of high fractions of PVA/CS, as well as small portions of GO, increases the tensile strength of the films. This reinforcing effect is attributed to the good GO sheets dispersion in the PVA/CS matrix, acting as a nanocharge that absorbs a good part of the force applied to the film. Also, it is due to the union between the amino groups of the CS and the carboxyl groups on the surface of the GO through covalent bonds and hydrogen bonds, thus contributing to the reinforcement of the films due to the formation of these molecular bonds [40].

#### 3.2.4. Degradation in a Simulated Biological Fluid 

##### Weight Loss

**Figure 7:**
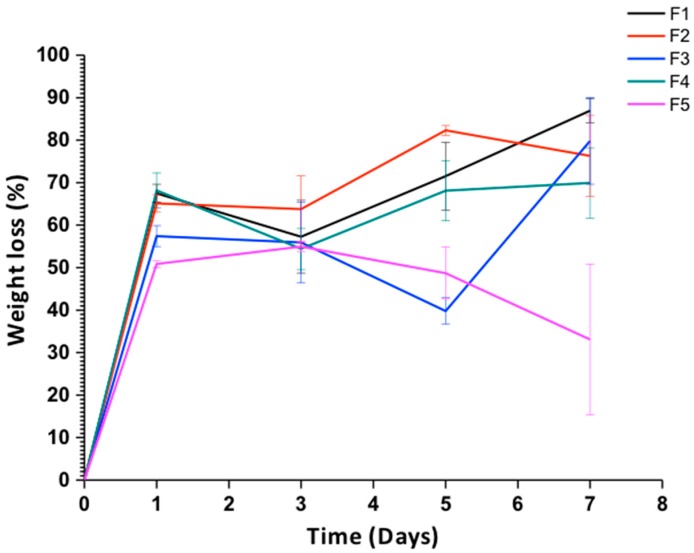
Weight loss vs. time. F1: CS/PVA/GO 20:80:0; F2: CS/PVA/GO 14.75:85:0.25; F3: CS/PVA/GO 19.5:80:0.5; F4: CS/PVA/GO 14.25:85:0.75 and F5: CS/PVA/GO 29:70:1. All the ratios are wt %. Data are shown as mean ± standard deviation of the mean (*n* = 3).

Figure 7 shows the results of the degradation percentage (weight loss) of the films with different proportions of GO during the seven days of immersion in SBF. The incorporation of GO in the polymeric matrix increased the stability of the films against SBF since the percentage of weight loss after seven days of immersion decreased from 86.95% for formulation F1 that did not contain GO up to 33% for the formulation F5 comprising 1% GO. This behavior is because the GO in the CS/PVA binary mixture gives the material a higher number of hydrogen bonds between the CS and the GO, which provide the material with high chemical stability in the polymer chains of the CS [41].

##### pH Changes

**Figure 8:**
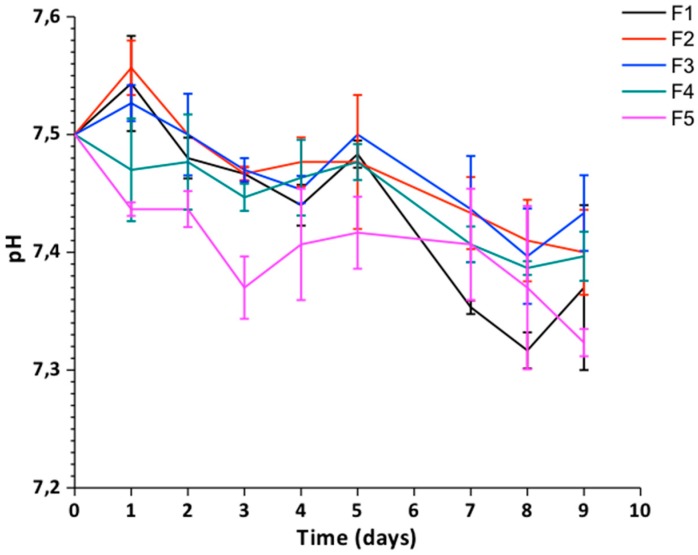
Study of the pH solution vs. immersion time in simulated body fluid (SBF) of the films. F1: CS/PVA/GO 20:80:0; F2: CS/PVA/GO 14.75:85:0.25; F3: CS/PVA/GO 19.5:80:0.5; F4: CS/PVA/GO 14.25:85:0.75 and F5: CS/PVA/GO 29:70:1. All the ratios are wt %. Data are shown as mean ± standard deviation of the mean (*n* = 3).

Figure 8 shows the pH changes in the SBF during the immersion period of the samples. The decrease observed in the pH when increasing the time of immersion of the samples in the SBF is associated with the attack to the amorphous zones of the polymers because they retain more acidic species and because they have greater susceptibility to degradation according to the research by Figueira-Maldonado et al. [42]. Besides, the detachment of the degradation by-products typical of CS such as glucosamine and *N*-acetylglucosamine contribute to said pH reduction, so that the formulations with higher CS content (formulations F1 and F5) show more significant reductions in the pH values. These by-products of CS degradation are found in the extracellular matrix of human tissue, making them harmless when released into the body [43]. The pH of the biological media is a fundamental constant for the maintenance of the vital processes since the enzymatic action, and the chemical transformations of the cells are carried out within a pH range between 6 and 8 [44,45,46]. All the formulations studied showed pH variations within the permissible range in the human body to promote life and maintenance of vital functions.

##### Scanning Electron Microscopy of the Films after Immersion in Simulated Body Fluid

**Figure 9:**
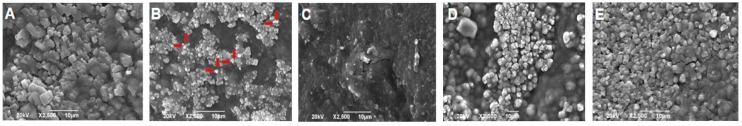
Morphology of CS / PVA / GO films after seven days in the degradation process in SBF. (**A**) F1: CS/PVA/GO 20:80:0; (**B**) F2: CS/PVA/GO 14.75:85:0.25; (**C**) F3: CS/PVA/GO 19.5:80:0.5; (**D**) F4: CS/PVA/GO 14.25:85:0.75; (**E**) F5: CS/PVA/GO 29:70:1.

Figure 9 shows SEM images of the CS/PVA/GO films after their immersion in SBF. The highly rough texture is due to the degradation process of the nanocomposite, which increases with GO content. Calcium phosphate deposition of a layer on the surface films can also be observed, which could be determined by energy dispersive spectroscopy (EDS) with a calcium composition of 36.70% and 16.4% phosphorus for formulation F2 (Supplementary Table S1). The deposition of said apatite layer on the surface (red arrows in Figure 9B) is suitable for a bioactive material since it promotes the interaction between the material and the surrounding tissue [47].

The morphological structure of the deposited layer presents typical morphological characteristics of apatite structures (globular structures), similar to those reported by different authors for materials with bioactive traits [48]. The morphology of the films before and after the immersion in SBF can be seen in Figure 5 and Figure 9, respectively, where an evident difference in the texture of the surface is noted.

#### 3.2.5. Antibacterial Activity

As shown in Table 3, no inhibition was observed for films that did not contain GO. Although some authors have demonstrated that chitosan shows activity against Gram-positive and Gram-negative bacteria, due to the loss of intracellular constituents [49,50], our results agree with other authors [51], which suggest that chitosan must be dissolved to observe antimicrobial effect, because PVA improves hydrogen bonds with chitosan, decreasing its solubility [52].

In the films with added GO, the results at 24 h of incubation showed that the addition contributes to completely inhibit the growth of the strains treated with 0.75% of GO and with a greater inhibition than 1.0%, an effect similar to what happens when essential oils are incorporated [53]. The strain with the least inhibition was *S. aureus*, which showed partial restraint at the concentration of 0.25% of GO, indicating that there is higher resistance by Gram-negative bacteria to inhibition due to CS/PVA effect. The GO effect suggests an interaction at the level of the cell membrane.

The dependence of the antibacterial activity on GO content, coincides with some investigations that show the GO has an inhibitory effect on the growth of Gram-positive bacteria, such as *S. aureus* [54,55], and Gram-negative, such as *E. coli* [54,56,57,58,59] and *Pseudomonas aeruginosa* [60], through damage to the cell membrane of bacteria when they come into contact with GO. Damage to the membrane can be caused by the sharp atomic edges of graphene, which can penetrate the cell membrane and physically disturb its integrity and also through lipid peroxidation induced by the oxidative nature of GO [61]. Oxidative stress is considered as an essential component of antimicrobial activity for bacterial cells exposed to GO [62].

#### 3.2.6. Biomodel Tests In Vivo

**Figure 10:**
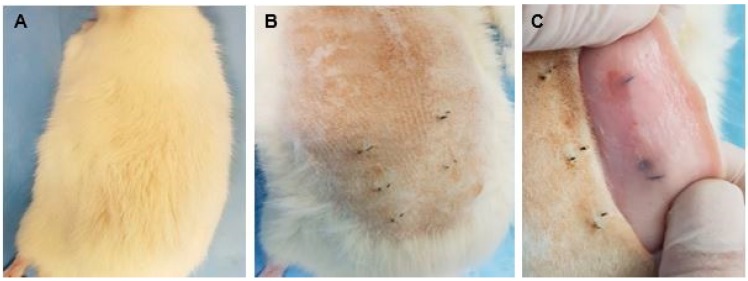
The dorsal area where the implantation was performed after 30 days. (**A**) Hair recovery; (**B**) absence of injuries and infections; (**C**) internal surface of the skin where the implanted samples encapsulated by scar tissue.

After 30 days of implantation in the biomodels, the recovery of the samples was performed, and in all cases, repair of the surgical defect created was observed. All the biomodels presented hair recovery (Figure 10A) and the absence of injuries and infections in the intervened areas with normal healing of the tissue architecture (Figure 10B,C). The material is initially compatible, an inflammatory reaction to a foreign body is apparent where the cells surround the fragments with a fibrous capsule and the rest of the soft tissues with regular appearance.

**Figure 11:**
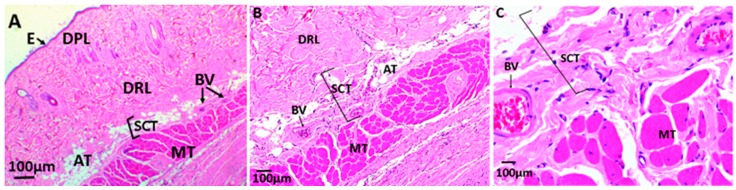
(**A**–**C**) Histological analysis of sample control. E: Epidermis; DPL: Dermis papillary layer; DRL: Dermis reticular Layer; BV: Blood vessels; AT: Adipose tissue; MT: Muscular tissue; SCT: Subcutaneous cellular tissue. (**A**) 4×; (**B**) 10×; (**C**) 40×.

**Figure 12:**
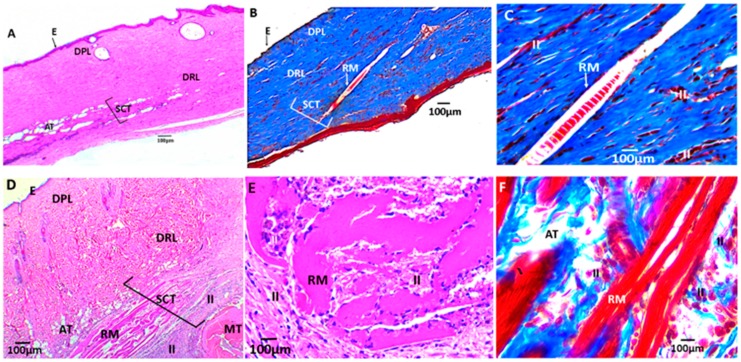
Histological analysis of experimental samples: (**A**–**C**) formulation F2 (CS/PVA/GO 14.75:85:0.25) and (**D**–**F**) formulation F3 (CS/PVA/GO 19.5:80:0.5). All the ratios are wt %. E: Epidermis; DPL: Dermis papillary layer; DRL: Dermis reticular layer; BV: Blood vessels; AT: Adipose tissue; H: Hypodermis; MT: Muscular tissue; SCT: Subcutaneous cellular tissue; RM: Remnant material; II: Inflammatory infiltrate. (**A**) 4×; (**B**) 10×; (**C**) 40×; (**D**) 4×; (**E**) 10×; (**F**) 40×.

**Figure 13:**
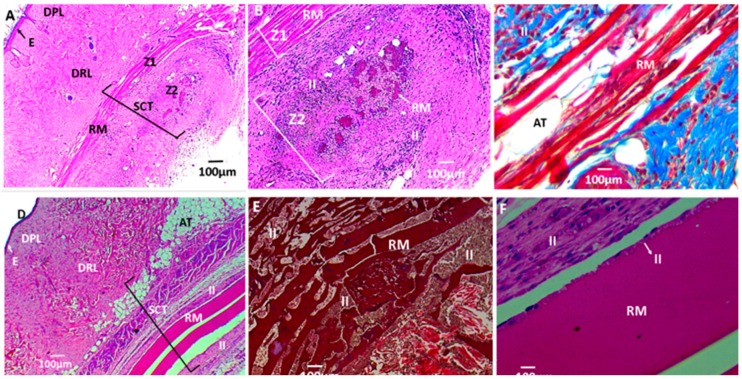
Histological analysis of experimental samples: (**A**–**C**) formulation F4 (CS/PVA/GO 14.25:85:0.75) and (**D**–**F**) formulation F5 (CS/PVA/GO 29:70:1). All the ratios are wt %. E: Epidermis; DPL: Dermis papillary layer; DRL: Dermis reticular layer; BV: Blood vessels; AT: Adipose tissue; H: Hypodermis; MT: Muscular tissue; SCT: Subcutaneous cellular tissue; RM: Remnant material; II: Inflammatory infiltrate; Z1: Zone 1; Z2: Zone 2. (**A**) 4×; (**B**) 10×; (**C**) 40×; (**D**) 4×; (**E**) 10×; (**F**) 40×.

Figure 11, Figure 12, and Figure 13 correspond to the histological analysis of the subcutaneous cellular tissue (SCT) of the biomodels (Wistar rats) with CS/PVA/GO films implanted. It is apparent that the control sample (porcine collagen) has been wholly reabsorbed and the healing is typical with a recovery of the tissue architecture (Figure 11A–C).

In the experimental formulation F2 (CS/PVA/GO 14.75:85:0.25) and F3 (CS/PVA/GO 19.5:80:0.5), normal healing is observed, and small film pieces remain with the presence of inflammatory cells surrounding them (Figure 12).

The samples with GO content at 0.25 and 0.5% showed more significant evidence of degradation, and less inflammatory response, the presence of inflammatory infiltrate being greater in the sample of F3(19.5CS/80PVA/0.5GO) (Figure 12A–C).

The formulations with 0.75 wt % and 1 wt % GO present less evidence of degradation and a greater inflammatory infiltrate. In Figure 13A,B, two zones of degradation are observed: Z1 with poorly degraded films and Z2 with highly fragmented films that are in the process of degradation by inflammatory cells. Figure 13C corresponds to zone Z1 at 40×.

Figure 13D–F correspond to films with a GO content of 1%. There is very little evidence of degradation, and at 40× the films surrounded by inflammatory cells are observed, starting the reabsorption at their edges.

The histological results show that the implanted materials are stimulating a typical foreign body reaction, which is the mechanism typically used by living beings to phagocytose subdermal implanted materials [63,64,65,66].

The degradation of the CS/PVA/GO films through fragmentation and phagocytosis is caused by cells belonging to an inflammatory infiltrate and their replacement with healthy tissue. Recovery of the standard architecture (Figure 12 and Figure 13), is an indication of a biocompatible material that eventually will be degraded entirely/reabsorbed by a foreign body reaction, with moderate GO content dependence as previously reported [25,67,68].

## 4. Conclusions

Films based on CS/PVA/GO nanocomposites were obtained, which showed suitable properties from the mechanical, chemical, and biological point of view for their application in tissue engineering.

The addition of GO to CS/PVA films generated an increase in tensile strength, surface roughness, and degradation time in both SBF and implanted in the biomodels. Also, the antimicrobial activity of the films against the bacteria studied, showed an increase with the content of GO, mainly because the mechanisms of inhibition of the bacterial growth of the GO are generated by contact between the GO and the cell membrane of the bacteria.

After SBF films immersion, a light layer of calcium phosphate was generated on the surface of the material, which is usually related to a bioactive material.

The films with 0.75 wt % of GO were the best formulation with complete growth inhibition of the tested bacteria and advanced degradation in vivo with low foreign inflammatory response of the tissue, demonstrating its potential for application in films for tissue engineering.

## Figures and Tables

**Table 1:** Formulations (F1–5) used for the preparation of nanocomposite solutions.

Component	F1	F2	F3	F4	F5
CS (wt %)	20	14.75	19.5	14.25	29
PVA (wt%)	80	85	80	85	70
GO (wt %)	0	0.25	0.5	0.75	1

CS: Chitosan; GO: Graphene oxide; PVA: Poly(vinyl alcohol).

**Table 2:** Interplanar distance of graphite and GO.

Material	Diffraction Peak (°)	Interlayer Distance (nm)
Graphite	26.46	0.336
GO	10.77	0.82

GO: Graphene oxide.

**Table 3:** Inhibition of CS/PVA/GO films against bacterial strains.

Strain	F1	F2	F3	F4	F5
*Bacillus cereus*	---	++	++	+++	+++
*Staphylococcus aureus*	---	++	++	+++	+++
*Salmonella spp*	---	--	++	+++	+++
*Escherichia coli*	---	++	++	+++	+++

(+++) Complete inhibition of the pathogen; (++) Weak inhibition of the pathogen; (--) Weak pathogen growth; (---) Complete growth of the pathogen. F1: CS/PVA/GO 20:80:0; F2: CS/PVA/GO 14.75:85:0.25; F3: CS/PVA/GO 19.5:80:0.5; F4: CS/PVA/GO 14.25:85:0.75; F5: CS/PVA/GO 29:70:1. All the ratios are wt %.

## Supplementary Materials

The following are available online at https://www.mdpi.com/2218-273X/9/3/109/s1, Table S1: Energy dispersive X-ray spectroscopy (EDS) results of the formulation F2.
