# Supplementary material for: Antimicrobial Films Based on Nanocomposites of Chitosan/Poly(vinyl alcohol)/Graphene Oxide for Biomedical Applications
Source: Biomolecules. 2019 Mar 18;9(3):109. doi: 10.3390/biom9030109 (PMC6468879; doi:10.3390/biom9030109)
Supplement: Supplementary file 1 [file biomolecules-09-00109-s001.pdf]

Biomolecules **2019**, *9*, 109; doi:10.3390/biom9030109

Supplementary Materials: Antimicrobial Films Based on Nanocomposites of Chitosan/Poly(vinyl alcohol)/Graphene Oxide for Biomedical Applications

Sebastián Ruiz [1], Julián Andrés Tamayo [1], Johannes Delgado Ospina[2], Diana Paola Navia Porras[2], Mayra Eliana Valencia Zapata [1], José Herminsul Mina Hernandez [1], Carlos Humberto Valencia [3], Fabio Zuluaga [4] and Carlos David Grande Tovar [5,*]

[1] Escuela de Ingeniería de Materiales, Facultad de Ingeniería, Universidad del Valle, Calle 13 No. 100-00, Santiago de Cali 760032, Colombia; sebastian.ruiz.londono@correounivalle.edu.co (S.L.); julian.tamayo@correounivalle.edu.co (J.T.); valencia.mayra@correounivalle.edu.co (M.V.); jose.mina@correounivalle.edu.co (J.M.)

[2] Grupo de Investigación Biotecnología, Facultad de Ingeniería, Universidad de San Buenaventura Cali, Carrera 122 # 6-65, 76001 Cali, Colombia; jdelgado1@usbcali.edu.co (J.D.); dpnavia@usbcali.edu.co (D.N.)

[3] Escuela de Odontología, Grupo Biomateriales Dentales, Universidad del Valle, Calle 13 No. 100-00, 76001 Cali, Colombia; carlos.humberto.valencia@correounivalle.edu.co

[4] Laboratorio SIMERQO, Departamento de Química, Universidad del Valle, Calle 13 No. 100-00, 76001 Cali, Colombia; hector.zuluaga@correounivalle.edu.co

[5] Programa de Química, Facultad de Ciencias, Universidad del Atlántico, Carrera 30 No. 8-49, Puerto Colombia 081008, Colombia

* Correspondence: carlosgrande@mail.uniatlantico.edu.co; Tel.: +57-5-385-22

Table S1. Energy dispersive X-ray spectroscopy (EDS) results of the Formulation F2.

Spectrum	In stats.	C (% atom)	O (% atom)	Na (% atom)	Mg (% atom)	P (% atom)	Cl (% atom)	Ca (% atom)	Total
1	Yes	6.32	43.49	2.55	0.74	16.32	0.94	29.65	100.00
2	Yes	15.70	41.21	4.74	-	9.46	10.53	18.36	100.00
3	Yes	10.62	39.46	5.53	-	13.89	3.05	27.44	100.00
4	Yes	8.19	40.11	1.72	0.67	15.66	2.94	30.71	100.00
5	Yes	7.14	35.69	1.29	0.64	16.41	2.12	36.70	100.00
6	Yes	33.38	11.64	13.02	-	-	41.19	0.77	100.00
7	Yes	-	45.29	10.08	-	11.07	11.40	22.16	100.00
